# Robotics and Aseptic Processing in View of Regulatory Requirements

**DOI:** 10.3390/pharmaceutics15061581

**Published:** 2023-05-24

**Authors:** Andrea Tanzini, Marco Ruggeri, Eleonora Bianchi, Caterina Valentino, Barbara Vigani, Franca Ferrari, Silvia Rossi, Hermes Giberti, Giuseppina Sandri

**Affiliations:** 1 Staubli Robotics, Staubli Italia S.p.A, Via Rivera 55, 20841 Carate Brianza, Italy; a.tanzini@staubli.com; 2 Department of Drug Sciences, University of Pavia, Viale Taramelli 12, 27100 Pavia, Italy; marco.ruggeri@unipv.it (M.R.); eleonora.bianchi04@universitadipavia.it (E.B.); caterina.valentino01@universitadipavia.it (C.V.); barbara.vigani@unipv.it (B.V.); franca.ferrari@unipv.it (F.F.); silvia.rossi@unipv.it (S.R.); 3 Department of Electrical, Computer and Biomedical Engineering, University of Pavia, Via Ferrata, 27100 Pavia, Italy

**Keywords:** nanomedicines, pharmaceutical processes, sterile manufacturing, automation, robotics, GMP, annex 1

## Abstract

Several nanomedicine based medicinal products recently reached the market thanks to the drive of the COVID-19 pandemic. These products are characterized by criticality in scalability and reproducibility of the batches, and the manufacturing processes are now being pushed towards continuous production to face these challenges. Although the pharmaceutical industry, because of its deep regulation, is characterized by slow adoption of new technologies, recently, the European Medicines Agency (EMA) took the lead in pushing for process improvements using technologies already established in other manufacturing sectors. Foremost among these technologies, robotics is a technological driver, and its implementation in the pharma field should cause a big change, probably within the next 5 years. This paper aims at describing the regulation changes mainly in aseptic manufacturing and the use of robotics in the pharmaceutical environment to fulfill GMP (good manufacturing practice). Special attention is therefore paid at first to the regulatory aspect, explaining the reasons behind the current changes, and then to the use of robotics that will characterize the future of manufacturing especially in aseptic environments, moving from a clear overview of robotics to the use of automated systems to design more efficient processes, with reduced risk of contamination. This review should clarify the regulation and technological scenario and provide pharmaceutical technologists with basic knowledge in robotics and automation, as well as engineers with regulatory knowledge to define a common background and language, and enable the cultural shift of the pharmaceutical industry.

## 1. Introduction

Innovation in drug therapy is mainly focused on biotech products and nanotechnologies are of paramount importance within that frame. In fact, many biotech nanomedicines recently reached the market thanks to the drive of the COVID-19 pandemic, and these innovative products aim at precision medicine, minimization of adverse effects/toxicity, and at meeting the previously unmet medical needs of patients [1,2].

There is no consensus about the definition of nanomedicine. In Europe, the European Medicines Agency (EMA) designates nanomedicine as “the application of nanotechnology in view of making a medical diagnosis or treating or preventing diseases” through exploiting the properties of materials at nanometer scale (approximately 0.2–100 nm) [3]. However, in the United States, the Food and Drug Administration (FDA) follows a more restrictive approach, considering both size (materials with nanoscale dimensions of approximately 1–100 nm) and function (whether physical or chemical properties or biological effects are attributable to dimensions up to 1000 nm) to determine whether a product involves nanotechnology [4]. On the basis of the recommendation of the European Commission, the term “nanomaterial” refers to a natural, incidental or manufactured material consisting of solid particles that are present, either on their own or as identifiable constituent particles in aggregates or agglomerates, and where 50% or more of these particles in the number-based size distribution fulfils at least one of the following conditions: (a) one or more external dimensions of the particle are in the size range from 1 nm to 100 nm; (b) the particle has an elongated shape, such as a rod, fiber or tube, where two external dimensions are smaller than 1 nm and the other dimension is larger than 100 nm; (c) the particle has a plate-like shape, where one external dimension is smaller than 1 nm and the other dimensions are larger than 100 nm [5].

**Figure 1:**
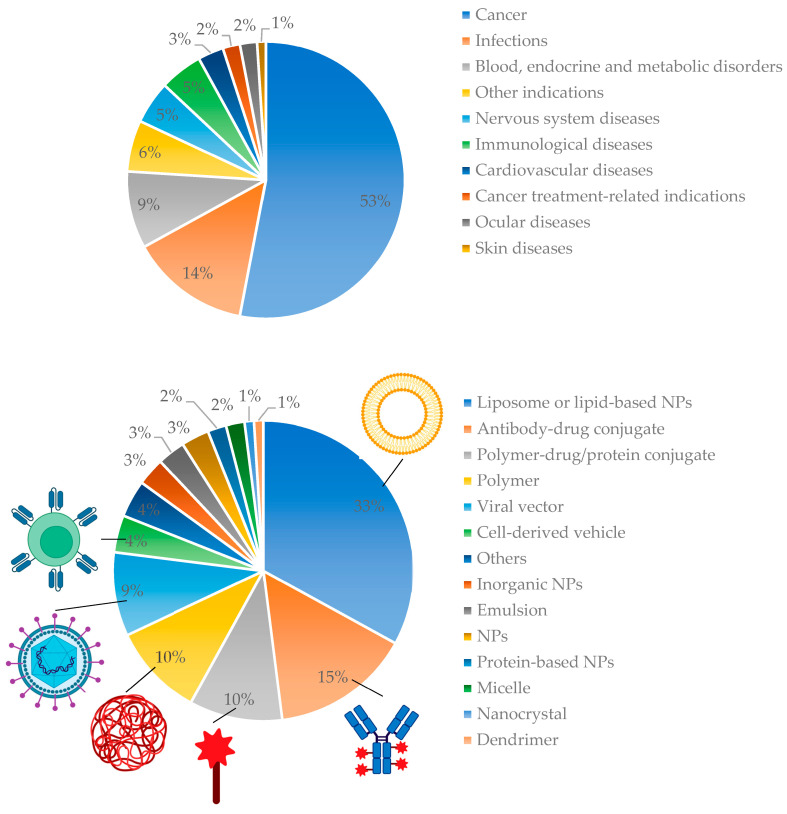
Overview of the nanomedicines that are available commercially or in clinical trial. Modified from [6].

Despite that, only 13 nanomedicines had been approved by the US FDA before 2015. However in 2021, 100 nanomedicines had been marketed, and another 563 new nanomedicines were under clinical trial or in other stages of drug development. The therapeutic areas involve the treatment of cancer (53%) and infections (14%), as well as others, such as blood disorders, and various diseases concerning endocrine and metabolic, the nervous system, immunological, cardiovascular, ocular, and the skin [6]. Moreover, innovative vaccination strategies are also based on nanomedicines. Among the different formulation types, liposomes or lipid-based nanoparticles (33%), antibody–drug conjugates (15%), polymer-drug–protein conjugates (10%), and polymeric nanoparticles (10%) are the most promising. Figure 1 summarizes the nanomedicines commercially available or in clinical trials [6,7].

Considering also that all these products are parenteral and according to the GMP are sterile, the costs associated with the manufacturing of nanomedicines are extremely high and significant efforts have been made to successfully develop unit operations capable of handling continuous stream processing, to increase product homogeneity and quality, and reduce production time [8]. Moreover, nanomedicines generally cannot be terminally sterilized but require an aseptic process to avoid medicine degradation. Ideally, the integration of all unit operations in a single manufacturing platform increases the production efficiency, and this achieves less expensive and safer drugs for the benefit of the patients [9]. The introduction of continuous manufacturing in the biopharmaceutical industry is a guarantee of high quality and high process efficiency and this type of process has been recently achieved, although only a limited number have been implemented.

**Figure 2:**
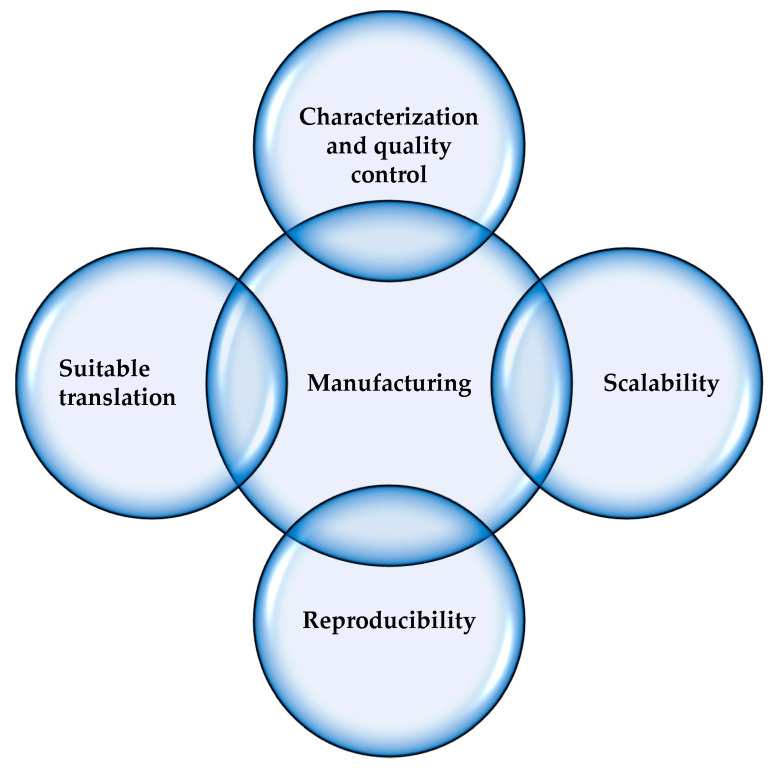
Challenges in the development and commercialization of nanomedicines. Inspired from [16].

In the manufacturing of sterile nanomedicines, there are many challenges to be overcome, and among these, the product and system complexity and the lack of real-time process information are the major ones, causing criticality in the batch scalability and reproducibility (Figure 2). Within this framework, there are a few examples in the literature that describe the rational development and manufacture of nanomedicines involving design of experiments (DoE) and process analytic technologies (PAT) to assure product quality and an efficient production process [10,11,12]. For these reasons, considering the decreased time to market, research and development expenditure, environmental impact, cost, and, above all, the increased product quality, the manufacturing processes are being pushed towards continuous production [13,14]. Regulatory agencies have pushed for the modernization of pharmaceutical manufacturing and use of continuous manufacturing to transform the pharmaceutical industry, facilitating the transformation from batch production to continuous production in the pharmaceutical field.

Continuous manufacturing is the integration of a series of operations to produce or process products into a single process. In continuous production, the raw materials and the products are continuously loaded, processed, and discharged without supply chain interruption. In addition, the idle time between the different stages is removed, human errors are eliminated, and employers do not require training on complex production processes. Companies can also focus their personal resources on development, testing, and maintenance rather than production [9,15].

**Figure 3:**
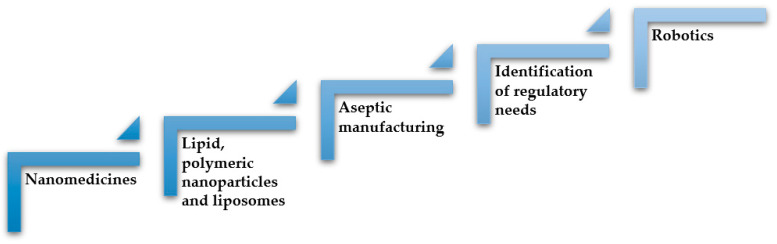
Uncertainties related to the nanomedicine regulatory process. Inspired from [17] with permission.

Therefore, continuous processes are more efficient than batch manufacturing in that they decrease the number of production stages and offer better flexibility to scale-up production. Machines can lead to higher product quality as well as more complete and reliable production, measurement data collection, and documentation. In the context of Pharma 4.0, the concept of continuous manufacturing embraces the use of robots, going beyond lower production costs and higher yields. Continuous processing and in particular robotics should cause a big change probably within the next 5 years, leading to an increase in process efficiency and quality. This paper aims at describing the regulation changes mainly in aseptic manufacturing and the use of robotics in the pharmaceutical environment to fulfil the requisites required by GMP (Good Manufacturing Practice), that are extremely important for biotechnological drug products and nanomedicines (Figure 3).

Therefore special attention will be paid first to the regulatory aspect, explaining the reasons behind the current changes, and then second to the use of robotics that will characterize the future of manufacturing especially in sterile environments, moving from a clear over-view of robotics to the use of automated systems to design more efficient processes, with reduced risk of contamination.

## 2. The Regulatory Framework

The pharmaceutical market is one of the rare markets where the consumer (in this case the patient), cannot usually choose which drug to be administered with. For this reason, the concept of quality and safety gains special importance, having a direct effect on patient health, likely when the health itself is compromised and actions are being taken to achieve recovery.

The GMP are included in the EudraLex volume 4 (The rules of governing medicinal products in the European Union, laid down in Commission Directives 91/356/EEC, as amended by Directive 2003/94/EC, and 91/412/EEC), released by the European Commission. GMP consists of a set of principles and guidelines to define the minimum requirements to guarantee drug quality and safety for human and veterinary use in Europe [18]. For these reasons, pharma industries should strictly adhere to these and periodically demonstrate their relevance through GMP inspections by European Regulatory Agencies, in particular the EMA (European Medicine Agency) [19].

### Annex 1–2022 Release—Quick Review

Annex 1 to GMP (released in August 2022) is the document that introduces the innovative vision of the pharma industries and gives all the tools for the process improvements using robotics, a technology well-established in other manufacturing sectors, that should lead to a big change in the pharma industries in a short time. Although this is mandatory for the European market, it could be a starting point for revisions of other regulations worldwide, given its cutting-edge approach [20].

The Annex 1 clearly states the scope of preventing contamination and cross-contamination in production not only with respect to sterile medicines, but also of medicines where sterility is not mandatory, focusing on QRM (Quality Risk Management) and CCS (Contamination Control Strategy). Annex 1 proposes a holistic approach for medicine manufacturing involving all the key areas in pharma, from the pharma quality system to quality control [21].

This is greatly underlined by the occurrence of specific key words throughout the documents, as reported in Table 1, and in particular the words “contamination” and “risk” are repeated more than 100 times.

Annex 1 focuses on manufacturing technologies as the state of art, and this is paired to a risk-based approach rather than only a quality assurance/control approach, forcing the rational design of each single production aspect, from the raw materials to the finished products.

Since contamination is a key point, particular attention is devoted to the technology suitable to reduce the risk of it happening.

**Figure 4:**
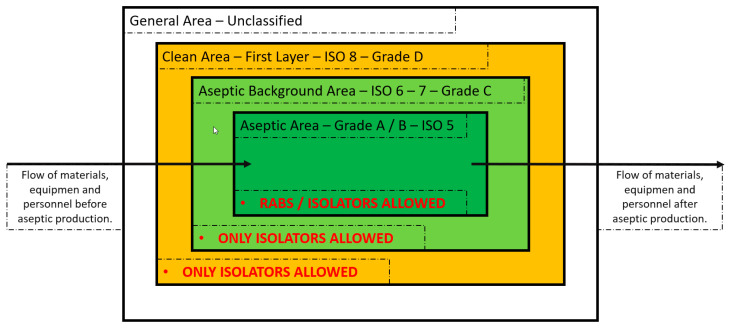
Schematic of the environmental classes and the technological features to maintain them on the basis of Annex 1–2022 release modified from [22] with permission.

Before Annex 1–2022 release, conventional clean rooms (class A environment) were always an acceptable choice. However, these were surrounded with rooms having a subsequent environmental class, from B to C (Figure 4). In this set up, separated rooms are mandatory, and the dressing procedures are gradual since each class is characterized by specific airborne particulate and microbiological limits, connected through airlocks (an intermediate chamber with two airtight doors or openings to permit passage between two dissimilar spaces). Moreover, in the clean rooms the drug products are directly in contact with the environment and with the personnel that could have been exposed to high potency active ingredients, potentially toxic.

Annex 1–2022 release addresses the manufacturer to barrier systems, such as RABS (restricted access barrier system) and isolators, as the preferred choice, and suggests that the use of conventional clean rooms should be adequately justified. Along with this, the concepts of automation and robotics are introduced as a further advancement to minimize contamination risk and personnel exposure.

**Figure 5:**
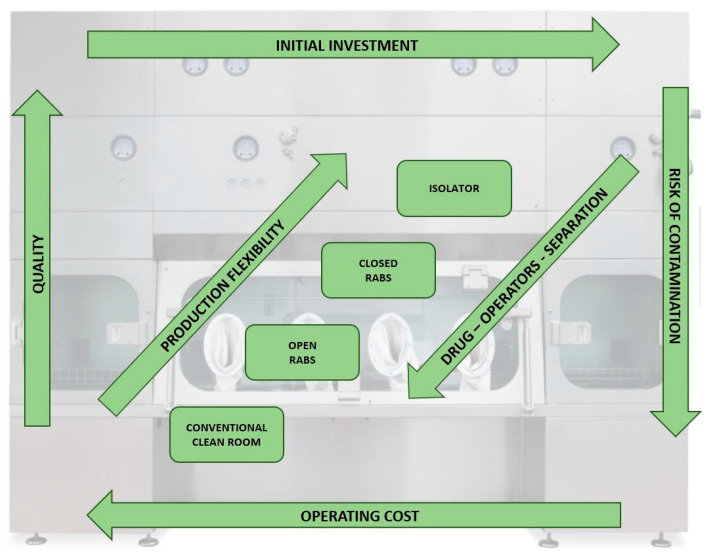
Chart that correlates quality and risk of contamination with investments for the conventional clean room, RABS, and isolators.

Among cleanrooms, RABS, and isolators, there are distinct structural and procedural differences that not only influence the level of quality assurance, flexibility, and strong reduction of contamination risk, but also differentiate the relative costs, addressing the initial investment and the operational cost of the plant (Figure 5).

Despite the concept of CCS being well established, Annex 1–2022 release requires the introduction of a holistic approach to control contamination risks, based not only on a planned set of controls on the environment and the products (microorganisms, pyrogens and particulates), but also on strong rationales regarding strategies to be adapted to control the risk of contamination.

## 3. Robotics

The origin of the term is related to the Czech writer Karel Čapek, who first used the word robot in 1920 in the novel entitled RUR (Rossumovi Univerzální Roboti-Universal Robots of Rossum), where an imaginary humanoid, identified as a robot appeared to invoke the Czech word “robota”, a literal term meaning “heavy labor”. The accepted definition of robot is any machine (anthropomorphic or not) capable of performing with different degrees of independence a job in place of a human [22]. In common usage, a robot is an artificial device that performs certain actions based on commands given, under direct human supervision, or autonomously based on general guidelines [23]. Robots are currently used for replacing or assisting humans in many fields, such as in manufacturing, construction, handling of heavy and hazardous materials, or in environments not compatible with human life, and furthermore are fundamental in a broad range of sectors, such as automotive, packaging, forging, and surgery/rehabilitation [24].

The robot usually is composed of two major components, the arm and the controller. The arm consists of the electromechanical part that performs the movement, and it is formed by a mechanical structure and drive (usually an electric motor) that sets the structure in motion [25]. The connection points of the mechanical part, where the motors are located, are called joints. Depending on the type of motion, joints are either rotary or linear.

The controller is the brain that sets the motor’s motion and calculates its movements, enabling the robotic arm to follow the desired trajectory and reach the assigned target in space and time. In addition to the robot there is the gripper, a tool that allows the robot to manipulate the objects and to complete its operations.

**Figure 6:**
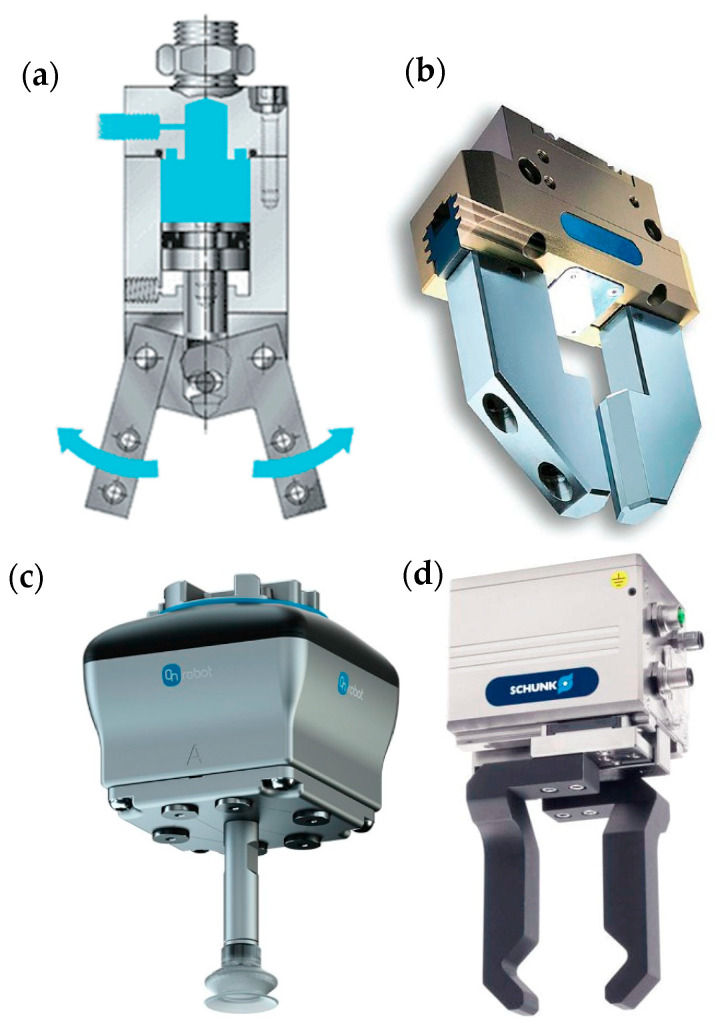
(**a**) Pneumatic gripper, (**b**) hydraulic gripper, (**c**) vacuum gripper, and (**d**) electric gripper; modified from [22].

Grippers can be classified in different groups depending on their mechanisms [26,27,28], as shown in Figure 6.

Mechanical grippers (Figure 6a) are based on mechanical interlocking while pneumatic grippers (Figure 6b) are based on elements moved using compressed air as actuators of “fingers” closing/opening. Hydraulic grippers (Figure 6c) work similarly to pneumatic grippers but using a fluid (usually oils) instead of compressed air. They are heavy-duty grippers using forces usually higher than 50 kg required for heavy handling. The presence of oil renders them critical for maintenance and the risk of environmental contamination. The vacuum grippers use suction cups that create a vacuum on the surface of the workpiece to be handled, allowing their manipulation, and electrical grippers are formed from a variable number of electric drives, to actuate the manipulation.

### 3.1. Robot Characteristics

Robot characteristics and performances are various and assume different importance depending on the task the robot is expected to perform.

Robot reach represents the maximum working radius that the robot can cover in space. It is conventionally measured in mm and indicates the distance from the center of the robot base to the center of the robot flange in the position of maximum extension. Depending on the type of model, this refers to the radius of circumference of the robot’s movement or the diameter (e.g., Delta robots) [29].

Robot payload indicates the maximum weight that the robot is able to move during its operations. This indicates the load liftable calculated in the center of the flange, but this is not an exhaustive evaluation. In fact, since the robot is expected to handle the gripper and the object itself, the center of mass of these two components cannot overlap to the center of the robot flange. Thus, the payload could decrease significantly depending on the distance from the object “gripper + part” center of mass and the robot flange [30].

Another fundamental point is the inertia of the moved object, that describes the weight distribution in space and therefore the moment of inertia on the axes of the robot during movements (carrying 10 kg condensed in one point is different from carrying 10 kg distributed along a 500 mm body).

**Figure 7:**
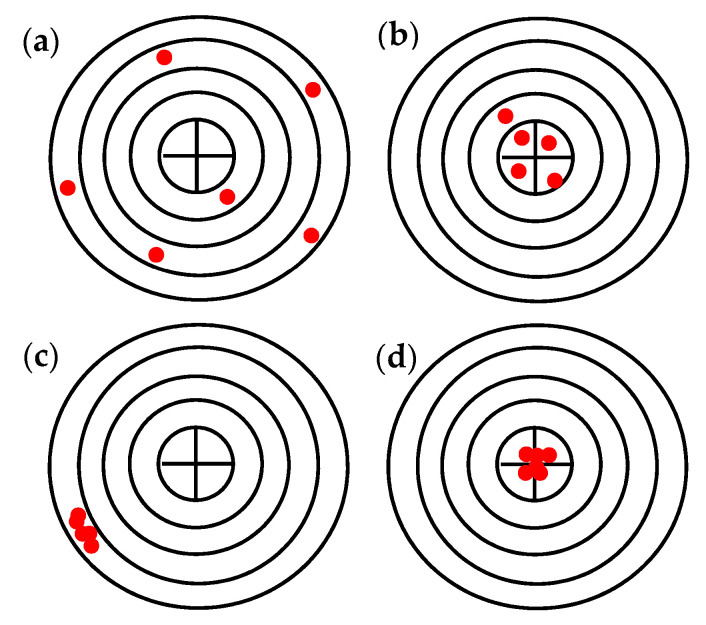
Schematic representation of the concepts of repeatability, accuracy, and visualization: (**a**) bad repeatability and bad accuracy, (**b**) bad repeatability and good accuracy, (**c**) good repeatability and bad accuracy, and (**d**) good repeatability and good accuracy.

Resolution, accuracy, and repeatability are key parameters that define the suitability of a robot for a particular operation. In particular, the resolution is defined as the measurement of the smallest deviation of the magnitude being measured by the sensors and therefore depends on the sensitivity of the sensors of the robot. The accuracy is defined as the maximum position error the robot performs in reaching a given assigned target point in space, while the repeatability defines and measures the robot’s ability to return to the same point in Cartesian space by always replicating the same motions [31,32]. The latter is the most significant parameter because traditional robotic applications are essentially designed to reach points in space in a repetitive way. In Figure 7 schematic representation of the concepts is reported.

Another important parameter is the speed, which can be divided into joint or flange center speed [33,34].

The joint speed indicates the rotational speed at which each individual joint can move, expressed in °/s or rad/s (with significant differences between nominal speed and maximum speed), while the flange center speed is the linear speed obtained at the center of the flange once the trajectory has been interpolated and translated from each individual joint. This is usually expressed in mm/s.

The parameters described above are the main decision-making elements (besides economic) for general industry applications. However, for challenging applications, where time and weight are critical there are other crucial parameters that should be considered.

### 3.2. Robot Types—Commercially Available Solutions

**Figure 8:**
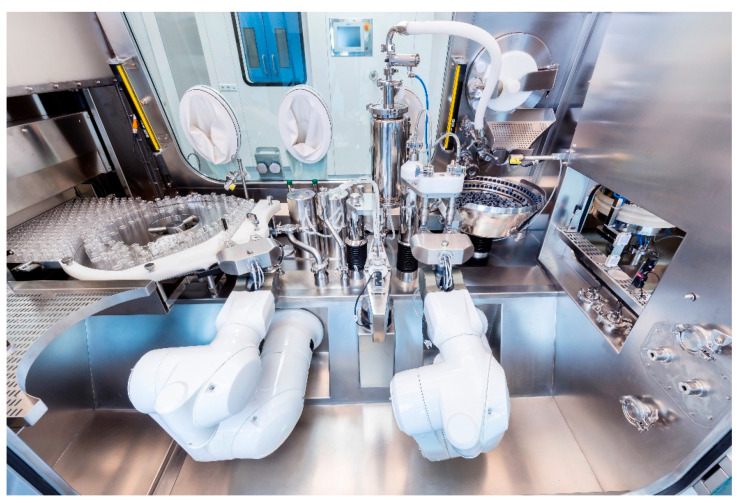
Example of robotic vial filling line (courtesy of Steriline).

Figure 8 reports the schematic of commercially available robots.

#### 3.2.1. Cartesian Robots

The Cartesian robot is a robot that moves in a straight line along a Cartesian coordinate system—the *x*, *y*, and *z* axes of the plane. It gets its name from the reference system introduced by the philosopher and mathematician Descartes using the coordinate method to highlight its movements along orthogonal axes. Its special distinction point is that it has only prismatic (linear) joints for principal movements; it can therefore move only in a straight line on the three axes [35,36].

Cartesian robots, which are easy to install and manage, are often considered a cheaper alternative to other types of robots. The most representative applications involve pick and place, assembly operations, machine tool operation, and arc welding, while they cannot be used in washing operations, as they are not waterproof. A key feature is adaptability, in that the strokes and dimensions of each axis can be customized to perfectly suit the application, with the velocities and payload being independent of each other [37].

#### 3.2.2. SCARA Robot

SCARA robot (Selective Compliance Assembly Robot Arm) is usually characterized by a horizontally moving arm and by a vertically moving grip. It consists of four axes: the first two are rotary, the third is linear in the *z* axis, and the fourth is rotary, allowing rotation of the transported parts. This type is widespread due to its flexibility being less rigid than Cartesian robots but more rigid than Delta and 6-axis robots. These are able to perform assembly, picking and unloading operations requiring a relatively small gripper with high accuracy and speed although they are slower than Delta robots but faster than 6-axis robots. They are especially used for the “pick and place” processes [38,39].

#### 3.2.3. Delta Robot

The Delta robot is a parallel robot and consists of three arms connected by universal joints at the base. The key concept of “classical” Delta robots is the use of parallelograms with motion restriction to pure translations of the end platform, allowing only movements along the *x*, *y*, and *z* axes usually with only rotation around the *z* axes. Recently, models boasting up to 5/6 degrees of freedom were released on the market, allowing complex movements of the carried part. Due to the type of construction and actuation, the delta robot allows high-speed movements, but among the robots listed here it is typically the least accurate [40].

#### 3.2.4. Anthropomorph Robots

The first anthropomorphic robot dates to the early 1970s, and it was designed and conceived to help and replace humans in dangerous stages of the assembly line. Anthropomorphic robots move in five or more axes, resembling the human arm both in feature and articulation All the joints of the anthropomorphic robot are rotational, perfectly imitating the movement of the human arm, allowing major flexibility in performing multiple tasks [41]. Depending on the structure they can have different payloads from a few kilos, with a few tens of centimeters of reach, up to a few tons, transportable for more than three meters of reach [42].

### 3.3. Critical Comparison between the Different Robot Types

Currently, robotic arms, especially anthropomorphic ones, are present in all the manufacturing sectors. Aeronautics, electronics, and automotive are the most representative. Food manufacturing, pharmaceutics, and surgery are emerging fields of robotic application. Despite their excellent degree of maturity, tremendous growth is expected. Depending on the robot type, it is possible to target different goals. In Table 2 the comparison of the robot types based on their characteristic parameters is reported [43].

Depending on the specific operation, robot selection should occur after verification of the characteristic parameters. These include the following aspects:

(1) Payload: analysis of the weight, load losses according to the center of mass, and verification of compliance with inertias.
(2) Reach: analysis of the movements that the robot (with gripper) needs to perform to realize the required cycle.
(3) Performance Verification: analysis of the required repeatability/accuracy.
(4) Cycle Time (speed): analysis of movements to efficiently perform the movement and the time required.

Simulation environments using specific software houses are able to consider all the variables and to guide the suitable selection. The simulation allows verification in 3D environments of the application feasibility and includes the 3D drawing of the environment which the robot has to move in and which the robot has to interact with, as well as the parts to be moved/the process to be carried out. Mathematical models are developed to accurately simulate the virtual movement of the robot [44].

## 4. Pharmaceutical Manufacturing and Robotics

Despite robotics and automation being mentioned several times throughout the Annex 1–2022 release, the concept of robotics is fairly new in the pharmaceutical sector, except in secondary packaging [45,46].

However, robot applications in pharma could involve the entire production chain, from API (active pharmaceutical ingredients) to final packaging and palletizing, and especially the activities in aseptic environments, such as filling. The benefits of automation with robotics are mainly related to HSE (health, safety and environment), quality, and production efficiency, among others [47,48].

In particular, in an aseptic environment the advantages related to the robot and automation are easily comprehensible (assuming no errors or failures—whose risk is minimal in a properly engineered application). In fact, the robot characteristics make them particularly effective to follow a defined SOP (standard of procedure), that is perfectly within the GMP scope—reducing the risk of human error.

The robots greatly reduce the impact of non-ergonomic or risky operations, preventing the operator from performing repeated operations and from exposure to highly potent compounds, especially in cleaning and decontamination procedures.

Moreover, robots avoid the (continuous) presence of the operator who is a major risk of contamination in the pharmaceutical environment, thus increasing the quality and the safety of production and significantly lowering the risk of contamination. Barrier systems allow the reduction of the risk of contamination also in the presence of the operator, while, in a cleanroom. Although the laminar flow reduces contamination, the medicines are still directly potentially exposed [49].

More specifically, in a barrier system (RABS) the robot is usually present in the grade A internal environment, while in the grade B surrounding environment the operator performs all the operations, enabling much more comfort for operation having less demanding gowning.

Robots allow the smart management of data, increasing the quality of the batch records, since all the robot operations are recorded in detail along with the corresponding environmental parameters and robot status including trajectories and movements in space, giving complete traceability along the process. This enables the avoidance of a time-consuming manual procedure with less robustness by the operator. In addition, by increasing the number of sensors of robots and grippers, big data related to CPP (critical process parameters) are produced for more in-depth analysis (applying machine learning and AI tools). This allows an early identification of drift trends and manufacturing nonconformities and their causes. In this way, CAPA (corrective and preventive actions) can be taken promptly, limiting the risk of losing control of production quality.

Of course, data integrity should be guaranteed following ALCOA (attributable, legible, contemporaneous, original, and accurate) principles as described in GMP-Annex 11 on Computerized Systems.

Furthermore, robots are able to increase efficiency in root cause analysis if a deviation is detected. Finally, a medium to high level of automation also allows 24/7 production continuity according to the market needs, and simultaneously guarantees monitoring of operative condition via computerized systems, thus ensuring quality robustness [50].

All these benefits are extremely convenient in the manufacturing of sterile medicines, and this is clearly stated in the new Annex 1 release (2022) that introduces a holistic approach of risk and contamination and paves the way to automation, forcing both the technological and cultural change required.

## 5. Robotics in Aseptic Manufacturing

The technological and cultural shifts driven by the Annex 1–2022 release are strongly entangled and mutually influenced. Quality, traceability, and process efficiency are greatly improved by robotics, however QRM (quality risk management) should guide the change. As clearly stated in the Annex 1–2022 release “*The manufacture of sterile products is subject to special requirements to minimize risks of microbial, particulate and pyrogen contamination. The following key areas should be considered: Facility, equipment and process design should be optimized, qualified and validated according to the relevant sections of the Good Manufacturing Practices (GMP) guidelines. The use of appropriate technologies (e.g., Restricted Access Barriers Systems (RABS), isolators, robotic systems, rapid microbial testing and monitoring systems) should be considered to increase the protection of the product from potential extraneous sources of particulate and microbial contamination such as personnel, materials and the surrounding environment, and assist in the rapid detection of potential contaminants in the environment and product.*” [19].

This concept is strongly reinforced and the Annex 1 states that “*where possible, the use of equipment such as RABS, isolators or other systems, should be considered in order to reduce the need for critical interventions into the Grade A zone and to minimize the risk of contamination. Robotics and automation of processes can also be considered to eliminate direct human critical interventions (e.g., dry heat tunnel, automated lyophilizer loading, sterilization in place)*”. In the perspective of the GMP, robotics in aseptic production therefore plays a key role in the demanded technology push, especially in grade A. If the barrier technologies are a preferential solution, where the space available is limited (e.g., solutions such as isolators turn out to be difficult for use in contexts different from green-field projects), the use of robotics is more flexible, since there is a variety of available choices, as seen in the previous paragraph. The best option should be chosen by a multidisciplinary team where members from different departments (quality assurance and quality control and engineering) discuss and agree with the features needed. Figure 8 reports an example of a robotic vial filling line.

### 5.1. Decisional Criteria

**Figure 9:**
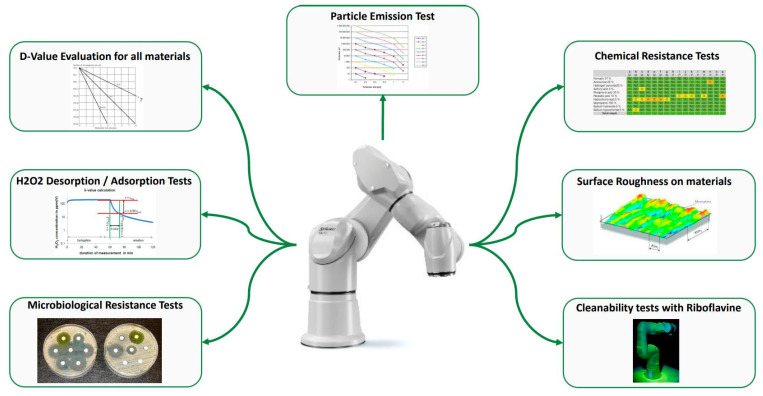
Schematic of the tests needed to understand the robot suitability to the environment.

Points such as particulate emission, microbial growth, and cross-contamination are clearly identified in the Annex 1–2022 release and should be considered in identifying a suitable robot type. Since the rationale of the Annex 1–2022 release is the reduction of risk of contamination, the robot should be compliant to GMP with particle emission, effective cleaning, and decontamination. These aspects need to be evaluated and must assume a particular focus in the CCS. Figure 9 reports the most important tests needed to understand the robot suitability to the environment.

#### 5.1.1. Particle Emission

Particle emission should be verified according to ISO 14644. This is mandatory to guarantee that the robot is not a source of particle contamination [51]. For this reason, the test is run in operations and depending on the results the robot receives an ISO n performance grade. This corresponds to the environmental grade that the robot can operate in. ISO5 (or better) classification is mandatory for classes A and B environments, ISO7 (or better) classification is required for Class C, and ISO8 (or better) is required for Class D.

#### 5.1.2. Surface Roughness

Roughness (Ra, expressed in μm) is the arithmetic mean value of the deviations (taken in absolute value) of the actual surface profile from the mean line. This measure is referred to a base length L of the analyzed profile to avoid the influence of other types of irregularities. It is characterized by the presence of spaced micro-irregularities on the surface texture, as high roughness acts as a point of particle and microorganism accumulation and provides a shelter for them during cleaning and decontamination [52].

As a de facto reference value, the Ra < 0.8 µm is the target for grade A environments (proposed by the European Hygienic Engineering and Design Group).

#### 5.1.3. Cleaning and Decontamination

It is crucial that the robot is composed of parts unable to promote microbial growth of occasionally deposited microorganisms. For these reasons the robot should be built using easy cleanable materials, largely inert to bacteria and mold proliferation. Basically, materials should not contain nutrients as microbial growth substrate and if inoculated with selected strains should not promote microorganism proliferation. The suitability rating is usually expressed as excellent (no growth substrate), good, low, and poor [53].

Moreover, since the cleaning and decontamination require chemicals (such as H_2_O_2_, isopropanol, hydrochloric acid, etc.), the materials should be resistant to these treatments. The resistance can be expressed both at the qualitative level (change of color, cracks, blistering, plaque) and at the quantitative level (changes in mass and number of defects) [54]. At the operational level, for cleaning, it is necessary to choose the chemical compatible with the robot and to adjust the frequency according to the compatibility degree.

A D-value should be attributable since it indicates the ease of surface decontamination. The D-Value expresses the time to achieve 6 Log reduction or in other words to kill 90% of the relevant microorganisms. All the materials should be considered identifying the critical one (based on a solid risk assessment), the worst case, and this allows a suitable time to obtain a complete decontamination to be set.

Moreover, the sorption of fumigation or vapor disinfection agents should be considered, and the K-value (constant of the Langmuir equation) assessed, to describe the material outgassing properties (time to reach 10% of the H_2_O_2_ concentration, indirectly describing the kinetics of H_2_O_2_ desorption).

However, cleaning is not only related to a specific material, but also to the constructed robot shape and surface roughness.

#### 5.1.4. Cleanability

Robot structure should facilitate cleaning and decontamination and should avoid hard-to-access accumulation points. The riboflavin test is recognized as the standard to assess cleanability. Since riboflavin is a fluorescent molecule, once it is applied onto a surface it is easy to detect fluorescent residues under UV light. So, if the cleaning process is not suitable the presence of fluorescence highlights it, maybe suggesting better options (among wiping, rinsing, etc.…).

### 5.2. Robot Suitability

All the features described in the above paragraphs do not provide an absolute answer, but rather they enable a holistic characterization to identify the specific robot that fits with the environment and the process.

At the grade A level, the Delta robot is excluded since it is designed to have a working area mainly below the robot body and therefore the laminar flow is interrupted above the production area. For these reasons, Delta robots are widely applied in secondary packaging, where the product is yet packaged. Cartesian, SCARA, or anthropomorphic robots are generally selected. However, they have different performances: Cartesian are used for extremely simple applications, SCARA are useful when the payload is limited in weight/inertia and requires simple manipulation, while the anthropomorphic possesses have extreme flexibility and redundancy in degrees of freedom, with widespread applications [55].

Operations in aseptic environments always polarize the choice toward anthropomorphic robots, in order to reduce the number of moving parts to the minimum. These guarantee speed, precision, and, most importantly, flexibility to the process. Moreover, whether the process is changed, the anthropomorphic robot allows ease of adaption with less constraints of the equipment design. In all cases the operating spaces should be carefully evaluated.

The gripper is of equal importance and QRM and CCS should be applied similarly as for the selection of the robot type choice. Ideally, the robot should be selected at the equipment or plant design stage.

In the case of revamping, a process nowadays faced by many industries to comply with the Annex 1–2022 release, the structural plant modification of layout can be burdensome and subjected to local laws, since the equipment and cleanrooms are already built, and robotics could reduce the risk of radical interventions [55].

The simulation is a key process to select and analyze the suitable robot/gripper types. In addition to 3D rendering, which enables the analysis of movements and trajectories at a glance, reliable verification of the operations should be achieved. Moreover robot speeds and accelerations should also be considered.

**Figure 10:**
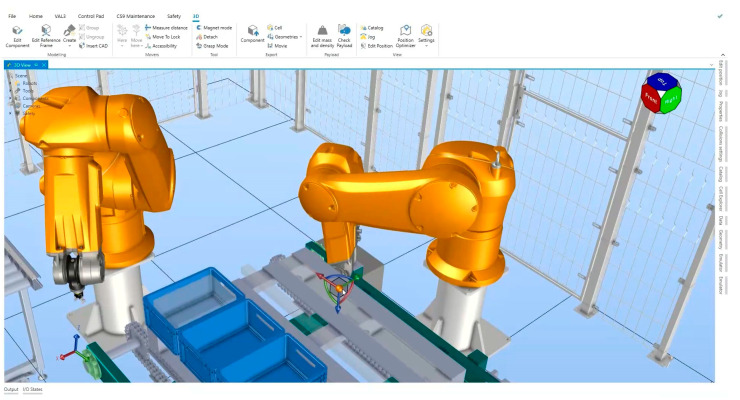
Simulation environment to analyze and visualize trajectories and equipment status forecast.

In addition, the preventive maintenance planning in accordance with CCS should be taken into account. Figure 10 reports an example of a simulation environment to analyze and visualize trajectories.

The simulation of the operation cycle with a 3D layout, including the robot, the gripper, and all the parts closely reflecting reality as much as possible, should give a maintenance estimation. This should allow reduction of both the risk of failure and downtime and the risk of batch rejection and should give economic benefit in terms of OEE (overall equipment effectiveness) and in terms of GMP compliance.

Simulation can also furnish the analysis of the unidirectional air flows present in the class A environments and this is important for the estimation of the suitability of the HVAC system. This is fundamental since if flow disruption occurs it is difficult to correct during the qualification processes.

**Figure 11:**
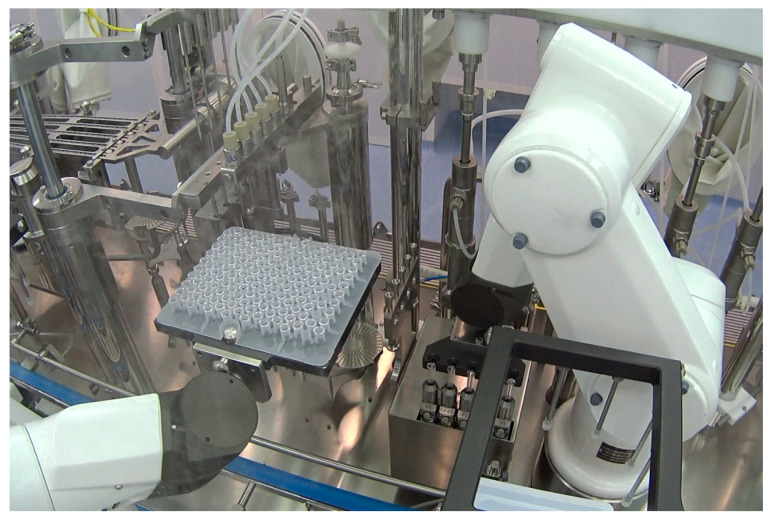
Smoke test study performed on a robotic open RABS filling line.

For example, in closed isolators, turbulent motion can be accepted, since the compliance with ISO classes and cleaning and decontamination reliability support almost zero risk of contamination (Figure 11).

Therefore, a robotic project for the pharmaceutical environment, especially for grade A, should definitely be carried out in a shared way by a multidisciplinary team involving different departments, from both the automation and the pharmaceutical industry, generating partnership among suppliers, OEMS, system integrators, and end users. An analysis focused on the pharmaceutical operation class, the choice of the specific robot and its design, the gripper design, and the type of installation represents a challenge that can be completely overtaken through a deep sharing of notions and ideas from one field to another. The pharmaceutical sector should help the robotics technicians to understand the requirements and the motivations peculiar to the pharmaceutical field, while, on the contrary, the automation companies should empower the pharmaceutical sector to understand the critical issues and the benefits of robots. The robotics and pharma worlds, especially aseptic manufacturing, recently started to deeply interact—a new way of working and new job figures capable of translating the languages spoken by the two worlds are emerging. The Annex 1–2022 release actually suggests a holistic approach where all stakeholders work together as a team with shared efforts toward a unique common goal, the increase of medicine quality and the guarantee of operator safety [55].

## 6. Conclusions

This review proves how the world of robotics can be considered as an ocean too big to navigate without guidelines acting like a compass for decisional steps. The variety of available commercial solutions and brands brings added value to specific process and application areas. However, the choice should consider the main and general strengths and weaknesses that characterize every kind of robot. A possible workflow for defining and choosing the parameters of automated cells has been shown here; this should enable the technical departments of pharmaceutical end users to work hands-on towards identifying and overcoming potential bottlenecks. Offline verification of feasibility and cycle time, together with robot simulations of maintenance need, have been shown as key steps in the workflow.

At the pharmaceutical level, the revolution is only beginning. Robotics is currently already widely used at the secondary packaging stage, but the coming years will see massive robot adoption in aseptic manufacturing environments as well. This implies both a technological shift, where robot manufacturers will be challenged to adapt to the requirements (type of robot, type of testing and supporting documentation), and a cultural shift, leading to automation and robotics-related decision-making processes.

The entry into force of the Annex 1–2022 release of GMP Volume 4 will characterize the next pharmaceutical industry revolution.

## Figures and Tables

**Table 1:** Key word occurrence in Annex 1 (2022) compared to the previous version.

Key Word Occurrence	Annex 1–2008	Annex 1–2022
Contamination	35	116
Risk (QRM)	20 (0)	100 (5)
CCS	0	51
Cleaning	6	29
Decontamination	1	18
Clean Room	11	46
Barrier	1	16
Isolator/Rabs	10	29
Technology/Technologies	6	27
Robotics	0	2

**Table 2:** Comparison of the robot types based on their characteristic parameters.

	Cartesian	SCARA	Delta	Anthropomorph
Reach	High	Small	Small	High
Payload	High	Low	Low–Medium	High
Repeatability	High	High	Medium	High
Velocities	Medium	High	High	Medium
Flexibility	Low	Medium	Low	High
